# Diverging Paths: Examining Initial Career Choices of Chief Residents in Emergency Medicine

**DOI:** 10.7759/cureus.94128

**Published:** 2025-10-08

**Authors:** Abagayle Bierowski, Erin Hoag, Casey Morrone, Ridhima Ghei, Kelly Kehm, Carlos Rodriguez, Peter J Tomaselli

**Affiliations:** 1 Emergency Medicine, Thomas Jefferson University, Philadelphia, USA; 2 Emergency Medicine, Wake Forest University, Winston-Salem, USA; 3 Emergency Medicine, NYU (New York University) Langone Hospital, Long Island, USA

**Keywords:** academic position, chief resident, choice of career, community position, emergency medicine, fellowship, fellowship training, residency program

## Abstract

Background and objectives: The career trajectories of emergency medicine physicians are thought to be shaped by various factors, including leadership roles during residency. Serving as a chief resident may be seen to be associated with a greater likelihood of pursuing an academic career, there are still important questions about the impact of this leadership experience unanswered. This study aimed to investigate whether serving as a chief resident increases the likelihood of pursuing academic or fellowship positions in the first post-residency job compared to peers who did not serve as chief residents.

Methods: Initial post-residency positions of 170 residents (35 chief residents, 135 non-chief residents) from a single academic emergency medicine residency program (2013-2024) were gathered and analyzed. Chi-square tests were performed to compare the frequencies of initial career choices (fellowship, academic, or community) between chief and non-chief residents, and effect sizes were calculated to determine the strength of the associations.

Results: Of 35 chief residents across 12 years, nine pursued a fellowship (25.71%), seven accepted an academic position (20%), and 19 obtained a community position (54.29%). Fifteen non-chief residents pursued a fellowship (11.11%), while 13 accepted an academic position (9.63%), and 107 obtained a community position (79.26%). Chief residents were statistically more likely to pursue fellowship positions (χ²: 5.19, *p* = 0.028) compared to non-chief residents. Though chief residents were twice as likely to accept an academic position compared to their non-chief resident colleagues, this result was not statistically significant (χ²: 3.14, *p* = 0.0766). Non-chief residents were statistically more likely to pursue community positions (χ²: 8.87, *p* = 0.0029).

Conclusions: While many factors shape career decisions, this study suggests that serving as a chief resident may increase the likelihood of pursuing a fellowship or academic position. Future research should explore the specific skills and experiences gained during chief residency that contribute to these career outcomes, and examine how residency programs can better support leadership development to optimize long-term career trajectories.

## Introduction

Career decisions following residency in emergency medicine shape long-term professional trajectories and influence the distribution of physicians across academic, fellowship, and community practice. These decisions are guided by multiple factors, including mentorship, exposure to academic environments, and leadership opportunities during training [1-3]. Serving as a chief resident is often regarded as a prestigious role that provides administrative, educational, and leadership experience, making it a potential driver of early career direction [4-6].

Within emergency medicine, limited data exist on the potential influence of this leadership role on early career trajectories. Prior studies across medical specialties have reported that serving as a chief resident is often associated with a higher likelihood of pursuing a fellowship, teaching faculty role, or academic position after graduation, although findings are not entirely consistent across the literature [2-10]. Moreover, prior studies rarely distinguish between fellowship training and direct entry into academic positions, leaving the relationship between these pathways and the chief resident role unclear.

This study sought to determine whether physicians who served as chief residents were more likely to pursue academic or fellowship positions in their first post-residency job compared with peers who did not serve as chiefs. We anticipated that chief residents would be more likely to enter academic or fellowship positions given the leadership experience, mentorship exposure, and professional visibility that accompany the role. 

This work was previously presented as an abstract at the 2025 Council of Emergency Medicine Residency Directors (CORD) Academic Assembly in Seattle, Washington, United States, in March 2025.

## Materials and methods

This study examined residency graduates from a single academic emergency medicine program, at Thomas Jefferson University Hospital in Philadelphia, Pennsylvania, United States, between 2013 and 2024 using a retrospective cohort design. During this time, the program graduated 171 residents, though one graduate was excluded from analysis because they pursued an additional residency program following completion of their training in emergency medicine. Of the 170 graduates analyzed, 35 served as chief residents and 135 did not. Chief residents were typically chosen in the second half of their second year in residency based on leadership potential, peer recognition (voting), and faculty/program leadership input.

The primary outcome was the graduate’s first post-residency position. Each graduate was categorized as entering a fellowship, accepting an academic faculty position at a teaching hospital without fellowship training, or beginning practice in a community setting. Distributions of initial career choice between chief residents and non-chief residents were compared using chi-square tests, with statistical significance defined as a p-value less than 0.05. Cramer’s V was also calculated to assess the strength of association, with values near 0.1 representing a small association, near 0.3 representing a medium association, and near 0.5 representing a large association.

We also conducted a post-hoc power analysis to evaluate whether our study was adequately powered to detect differences in academic faculty appointment and fellowship pursuit. Using two-sample tests of proportions with a two-sided α of 0.05, we calculated both the observed power at the empiric proportions and the minimum detectable effect size (MDE) required to achieve 80% power given the observed sample sizes.

## Results

**Figure 1:**
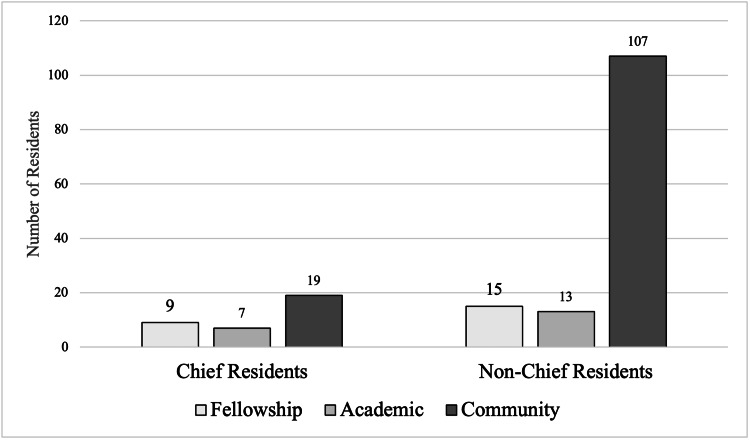
Visual distribution of fellowship, academic, and community positions among chief residents and non-chief residents

Among the 35 chief residents, nine (25.71%) pursued fellowship training, seven (20%) accepted academic faculty positions, and 19 (54.29%) began work in community practice. Among the 134 non-chief residents, 15 (11.11%) pursued fellowship, 13 (9.63%) accepted academic faculty positions, and 107 (79.26%) began work in community practice (Figure 1, Table 1).

**Table 1:** Comparison of first post-residency career positions between chief residents and non-chief residents

Initial Career Path	Chief Residents (n = 35)	Non-Chief Residents (n = 135)	p value	χ²	Cramér’s V
Fellowship	9 (25.71%)	15 (11.11%)	0.023	5.19	0.175
Academic	7 (20%)	13 (9.63%)	0.077	3.14	0.136
Community	19 (54.29%)	107 (79.26%)	0.003	8.87	0.228

Chief residents were more statistically likely to pursue a fellowship compared with their non-chief peers (25.71% vs 11.11%; χ²: 5.19, p = 0.023). Non-chief residents were significantly more likely to enter community practice compared with chiefs (79.26% vs 54.29%; χ²: 8.87, p = 0.003). Chiefs were also twice as likely as non-chiefs to accept an academic faculty position, although this finding did not reach statistical significance (20% vs 9.63%; χ²: 3.14, p = 0.136). To contextualize this null result, a post-hoc sensitivity analysis indicated that, with the observed sample sizes and α=0.05 (two-sided), an absolute difference of approximately 20.5 percentage points (≈30.2% vs 9.63%) would be required to achieve 80% power; the observed difference falls below this threshold, suggesting limited power to detect smaller effects. A year-by-year breakdown of initial post-residency position choices is presented in Table 2.

**Table 2:** Year-by-year distribution of initial career paths for chief and non-chief emergency medicine residency graduates (2013–2024) *This year excluded one resident who pursued an additional residency following completion of her emergency medicine training. +In 2013, the program appointed two chief residents, while in all subsequent years three chief residents were selected annually.

Year	Chief Residents			Non-Chief Residents		
	Community (n=19)	Academic (n=7)	Fellowship (n=9)	Community (n=107)	Academic (n=13)	Fellowship (n=15)
2024	0	2	1	10	1	3
2023	1	1	1	9	3	2
2022	2	0	1	10	1	1
2021	1	0	2	10	1	3
2020*	2	1	0	10	3	1
2019	2	1	0	9	0	1
2018	1	2	0	8	0	1
2017	2	0	1	7	1	1
2016	3	0	0	8	2	1
2015	0	0	3	8	0	1
2014	3	0	0	8	1	0
2013^+^	2	0	0	10	0	0

## Discussion

This study examined the relationship between serving as a chief resident and early career choices among graduates of a single academic emergency medicine program. Our findings suggest that chief residents may be more likely to pursue fellowship training immediately after residency and may also be more likely to enter academic positions, although this latter association did not reach statistical significance.

To our knowledge, this is the first study in emergency medicine to differentiate between fellowship training and immediate entry into academic positions as distinct post-residency career choices. This distinction is important, as fellowship training may serve as an additional pathway into academics but does not guarantee an academic appointment. Future research should expand upon this approach by examining not only first post-residency positions but also subsequent career choices following fellowship completion. Such analyses would provide valuable insight into whether fellowship-trained graduates are more likely to transition into academic careers or leadership roles compared with their peers, thereby clarifying the longitudinal impact of both chief residency and fellowship training on professional trajectories.

The increased likelihood of fellowship pursuit among chiefs may reflect several influences. Chief residents typically receive structured leadership and administrative experience that may foster interest in further specialized training [11]. The role may also increase exposure to faculty mentorship and institutional resources that can facilitate fellowship applications. Alternatively, residents who are already inclined toward leadership or academic careers may be more likely to seek or be selected for the chief position. Because the role is not randomly assigned and selection is based in part on faculty perceptions of leadership potential and academic inclination [12], the possibility of selection bias must be considered. This limitation reduces the ability to draw causal inferences, and the study findings should be interpreted as reflecting both pre-existing inclinations and the additional opportunities afforded by the chief role.

In addition to selection bias, other unmeasured factors may have contributed to the observed associations. Career trajectories are influenced by a range of variables, including gender, program year, mentorship exposure, and prior scholarly activity, which we were unable to systematically capture in this study. These potential confounders may influence both the likelihood of being selected as a chief resident and subsequent career decisions, complicating the interpretation of the observed relationships. Future studies incorporating larger, multi-institutional cohorts and controlling for these variables through multivariable analyses would provide a more robust assessment of whether the chief role independently predicts fellowship or academic career choice.

In considering these findings, it is also important to note that our residency program follows a three-year format. Prior research comparing three- and four-year emergency medicine programs has demonstrated that graduates of four-year programs may be more likely to pursue fellowship training and academic careers than their three-year counterparts [13]. One study that examined a transition from a three- to four-year format found that graduates of the four-year curriculum were more likely to secure non-fellowship academic appointments, assume leadership positions immediately after graduation, and publish scholarly work during residency [14]. These differences suggest that program length and structure may influence early career choices, and our results should be interpreted within the context of a three-year training model.

These findings should also be interpreted within the broader context of resident career decision-making. Post-residency career choices are shaped by a complex interplay of personal, professional, and financial factors, in addition to program-level influences. Residents have described career decision-making as guided not only by individual interests and values but also by mentorship, family input, and exposure to diverse career paths during training [15]. This highlights that the decision to pursue fellowship, academics, or community practice cannot be attributed solely to holding a chief resident position. Rather, the chief role represents one of many factors that may shape early career choices, and its influence likely interacts with broader processes of mentorship, reflection, and opportunity.

It is also notable that the majority of our graduates, including many who served as chief residents, entered community practice. This highlights that the leadership skills developed during the chief resident year have broad applicability. The administrative, educational, and organizational competencies fostered during this time are not limited to academic environments but are transferable to a wide variety of practice settings [16]. Residency programs may benefit from intentionally structuring the chief year to cultivate leadership skills that prepare graduates for success regardless of career path [17,18].

Limitations

This study has several limitations. It was conducted at a single institution and may not be generalizable to all residency programs. The retrospective design limits the ability to control for unmeasured factors such as personal preference, mentorship experiences, or financial considerations that may have influenced career choice. The relatively small sample size limited statistical power, particularly in detecting differences in academic career outcomes. For academic faculty positions, the observed power was low, and the minimum detectable effect size was large, raising the possibility that the non-significant result reflects insufficient power rather than a true absence of association; these findings should therefore be interpreted cautiously, particularly with respect to causal inference. The analysis focused only on the first post-residency position and does not capture long-term career trajectories. Although one graduate was excluded for pursuing an additional residency, other unique career paths may not have been fully accounted for.

## Conclusions

Serving as a chief resident was associated with a higher likelihood of pursuing fellowship training and showed a trend toward academic career choice, although most graduates from both groups at our institution ultimately pursued community practice. These findings suggest that the influence of the chief role on early career decisions is nuanced and shaped by multiple factors. Future multi-institutional studies should explore how leadership experiences during residency affect both early and long-term career outcomes, and how programs can best support leadership development for all trainees.
